# Survival Determinants and Sociodemographic Disparities in Early-Onset Non–Small Cell Lung Cancer

**DOI:** 10.1001/jamanetworkopen.2025.37307

**Published:** 2025-10-13

**Authors:** İrem Kar, Farheen Vhora, Maroun Bou Zerdan, Kubra Canaslan, Dhiksha Balaji, Badi El Osta, Sweta Balaji, Katie O’Reilly, Ticiana Leal, Tarun Jain, Suresh Ramalingam, Conor E. Steuer, Jennifer W. Carlisle, Selen Bozkurt, Fatemeh Ardeshir-Larijani

**Affiliations:** 1 Department of Biomedical Informatics, Emory University, Atlanta, Georgia; 2 Department of Biostatistics, School of Medicine, Ankara University, Ankara, Türkiye; 3 Department of Medicine, B. J. Medical College, Ahmedabad, India; 4 Winship Cancer Institute, Department of Hematology and Oncology, Emory School of Medicine, Atlanta, Georgia; 5 Department of Medical Oncology, Dokuz Eylul University, Izmir, Türkiye; 6 Aflac Cancer and Blood Disorders Center of Children’s Healthcare of Atlanta, Atlanta, Georgia; 7 Center for Ethics, Emory University, Atlanta, Georgia

## Abstract

**Importance:**

Patients with early-onset non–small cell lung cancer (NSCLC) represent a distinct and relatively understudied population. Identifying factors associated with survival is critical to guiding early detection and treatment strategies.

**Objective:**

To evaluate demographic, clinical, and socioeconomic factors associated with overall survival (OS) in early-onset NSCLC.

**Design, Setting, and Participants:**

This retrospective cohort study obtained data from the Surveillance, Epidemiology, and End Results database and included patients aged 18 to 50 years diagnosed with NSCLC between January 2010 and December 2021. Data analysis was performed between January 1 and March 31, 2025.

**Exposure:**

Early-onset NSCLC.

**Main Outcomes and Measures:**

The primary outcome was OS. Cox proportional hazards regression and Shapley additive explanations–enhanced machine learning models were used to improve interpretability and inform targeted interventions. Random survival forest models were used to identify factors associated with OS and assess model performance.

**Results:**

Among 18 595 patients with early-onset NSCLC, 9929 (53.4%) presented with stage IV disease. Most patients (15 597 [83.9%]) were aged 40 to 50 years (mean [SD] age, 44.6 [6.0] years), and 9710 (52.2%) were female. In terms of race and ethnicity, 2338 patients (12.6%) were Hispanic; 100 (0.5%), American Indian or Alaska Native; 2229 (12.0%), non-Hispanic Asian or Pacific Islander; 2693 (14.5%), non-Hispanic Black; 11 162 (60.0%) non-Hispanic White; and 73 (0.4%), unknown. In multivariable Cox proportional hazards regression, stage IV disease (hazard ratio [HR], 17.47; 95% CI, 15.28-19.96), liver metastases (HR, 1.45; 95% CI, 1.35-1.54), low household income (HR, 1.45; 95% CI, 1.33-1.58), squamous cell carcinoma histology (HR, 1.42; 95% CI, 1.33-1.51), and male sex (HR, 1.19; 95% CI, 1.14-1.25) were independently associated with worse survival. The random survival forest model demonstrated higher discrimination (C-index 0.765; 95% CI, 0.759-0.771), comparable to the Cox proportional hazards model (C-index, 0.774; 95% CI, 0.769-0.779). Feature importance analysis identified overall stage, radiation therapy, time to treatment, and household income as the most influential factors associated with survival. Among patients with stage I disease, rural residence was associated with a 65% increased risk of death (HR, 1.65; 95% CI, 1.23-2.08) and low household income was associated with a 96% increased risk of death (HR, 1.96; 95% CI, 1.52-2.55).

**Conclusions and Relevance:**

In this cohort study of 18 595 patients with early-onset NSCLC, most patients were diagnosed at advanced stages. Modifiable factors, including household income, residence, and time to treatment, were associated with survival outcomes. These findings support the need for increased awareness, early detection strategies, and efforts to reduce socioeconomic disparities in adults with NSCLC.

## Key Points

**Question:**

What factors are associated with overall survival in early-onset non–small cell lung cancer (NSCLC) in patients aged 18 to 50 years?

**Findings:**

In this cohort study of 18 595 adults with early-onset NSCLC, overall survival differed significantly based on stage at diagnosis and modifiable factors, including time from diagnosis to treatment, household income, and residential setting.

**Meaning:**

These findings suggest the need for earlier detection and targeted efforts to reduce socioeconomic disparities in adults with early-onset NSCLC.

## Teaser

This cohort study identifies demographic, clinical, and potentially modifiable socioeconomic factors associated with overall survival among adults aged 18 to 50 years who are diagnosed with non–small cell lung cancer.

## Introduction

Lung cancer remains the leading cause of cancer-related death in the US, with non–small cell lung cancer (NSCLC) accounting for approximately 85% of incident cases and 89% of prevalent cases.^1^ Early-onset NSCLC represents a small, yet distinct group, constituting 5% to 10% of cases.^2^ While the precise definition of early-onset NSCLC remains inconsistent across studies, an age cutoff of younger than 50 years is frequently adopted.^3,4^ Despite relatively small numbers, this population exhibits unique characteristics, such as higher prevalence of female, racial and ethnic minority, and nonsmoking patients and increased rates of actionable driver mutations.^5,6^

Young adults with NSCLC generally have fewer comorbidities and potentially better outcomes than older patients; however, conflicting survival data underscore the need to identify the factors that influence prognosis in this age group.^3,7^ Recognizing the growing public health impact, the National Cancer Institute introduced the Early-Onset Cancer Initiative to better understand and respond to cancers diagnosed at younger ages.^8^

While biological mechanisms such as accelerated aging^9^ may contribute to early-onset cancer, emerging evidence suggests that socioeconomic status (SES) is a critical determinant of disease outcomes. A meta-analysis and mendelian randomization study found that individuals from lower socioeconomic backgrounds had a 25% higher risk of developing lung cancer.^10^ In the Nordic countries, higher incidence rates of lung cancer have been consistently observed among individuals with lower SES, with notable variation across histological subtypes.^11^ Similarly, an Italian cohort study reported marked inequalities in lung cancer diagnosis, treatment, and mortality associated with socioeconomic factors.^12^ In Asia, environmental exposures combined with economic limitations have been linked to increased lung cancer risk in younger populations, reinforcing the importance of regional and contextual considerations.^13^ Beyond incidence and diagnosis, SES also influences access to advanced cancer treatments. For instance, patients from lower socioeconomic or racial minority groups often have reduced access to immune checkpoint inhibitors, next-generation sequencing,^14,15^ and other life-extending therapies, further exacerbating survival disparities.^16^

The objective of this study was to identify demographic, clinical, and potentially modifiable socioeconomic factors associated with overall survival (OS) among adults aged 18 to 50 years who are diagnosed with NSCLC. By improving understanding of the prognostic factors associated with outcomes in this understudied population, our goal was to inform more personalized and equitable approaches to care.

## Methods

### Data Source

This retrospective cohort study followed the Strengthening the Reporting of Observational Studies in Epidemiology (STROBE) reporting guideline and used data from the Surveillance, Epidemiology, and End Results (SEER) database (November 2023 release), which includes 17 cancer registries covering approximately 26.5% of the US population. We analyzed NSCLC cases diagnosed between January 2010 and December 2021.^17^ The study was approved by the institutional review board of Emory University, which waived the need for informed consent owing to the use of deidentified, publicly available SEER data.

### Study Population

We included patients aged 18 to 50 years who had been diagnosed with NSCLC between 2010 (the first year SEER reported stage information) and 2021. Cases were identified using *International Classification of Diseases for Oncology, 3rd Edition*, topography codes C340 to C343, C348, and C349 and histology codes 8010, 8012, 8013, 8020, 8046, 8050 to 8052, 8070 to 8078, 8140, 8141, 8143, 8147, 8250 to 8255, 8260, 8310, 8430, 8480, 8481, 8490, 8560, and 8570 to 8575. Inclusion criteria were (1) microscopically confirmed malignant histology and (2) age at diagnosis of 50 years or younger. We excluded cases with missing survival or treatment data or nonmalignant histology. A diagram summarizing case selection is provided in eFigure 1 in Supplement 1. To reduce selection bias, all eligible cases within the study period were included to ensure a representative and generalizable cohort.

### Study End Points and Covariates

The primary outcome was OS, defined as the time in months from diagnosis to death. Demographic, SES, and clinical variables included age (18-29, 30-39, or 40-50 years), sex (female or male), race and ethnicity (Hispanic, non-Hispanic American Indian or Alaska Native, non-Hispanic Asian or Pacific Islander, non-Hispanic Black, non-Hispanic White, or unknown), household income (<$55 000, $55 000-$75 000, $75 000-$89 999, or ≥$90 000), and rural or urban residence. Race and ethnicity data were collected because they are relevant to examining sociodemographic disparities in NSCLC outcomes. Median household income was an area-based variable derived from the zip code tabulation area of the patient’s residence at diagnosis. Clinical factors included stage at diagnosis (stages I-IV, based on the Derived American Joint Committee on Cancer–7 Stage Group); presence of bone, brain, or liver metastases; histological subtype (adenocarcinoma, large cell carcinoma, squamous cell carcinoma, or other or unspecified); and time from diagnosis to treatment. Treatment modalities included surgery, radiation therapy, and chemotherapy. Metastatic disease was represented using separate binary indicators for bone, brain, liver, and lung involvement. This approach maintains site-specific clinical interpretability and avoids potential multicollinearity that may arise from composite variables such as total number of metastatic sites or oligo-organ disease definitions.

### Statistical Analysis

Data were analyzed between January 1 and March 31, 2025. Data extraction and preliminary survival analyses were conducted using SEER*Stat, version 8.3.6.1.^18^ All statistical modeling and visualizations were performed using R, version 4.4.1 (R Program for Statistical Computing), and Shapley additive explanations (SHAP) analyses were conducted in Python, version 3.12.7 (Python Software Foundation). All study code is publicly available on GitHub.^19^

Descriptive statistics were used to summarize demographic, clinical, and treatment-related characteristics. Histological subtype was classified as adenocarcinoma, squamous cell carcinoma, large cell carcinoma, or other or unspecified. Treatment variables were included in the models as separate binary indicators (yes vs no or unknown) for surgery, radiation therapy, and chemotherapy. Treatments not recorded in the SEER database, such as immunotherapy, were excluded from the analysis. We excluded patients with missing survival or treatment data to ensure accuracy of outcome classification and treatment exposure. While this complete-case approach may introduce selection bias, the large sample size reduces concerns about generalizability, and imputation was not feasible due to limited auxiliary data on missingness.

Kaplan-Meier survival curves were generated to visualize differences in OS across key categorical variables, including age, sex, household income, rural-urban status, race and ethnicity, stage, and histology. The log-rank test was used to evaluate differences between groups. The survminer R package was used for Kaplan-Meier curve estimation and visualization.^20^

Period survival analysis^18^ was used to estimate conditional survival probabilities at 1-, 2-, 3-, 4-, and 5-year intervals. Univariable and multivariable Cox proportional hazards regression analyses^21^ were conducted to estimate hazard ratios (HRs) with 95% CIs. Variables with clinical relevance and statistical significance in univariable analysis were included in the multivariable model. To address multicollinearity, correlated variables such as TMN stages were excluded in favor of the overall stage variable. Statistical significance was defined at 2-sided *P* < .05. The survival package in R was used to fit Cox proportional hazards models.^22^ The initial model included 18 variables. After removing the 3 staging variables (TNM) due to collinearity and excluding additional variables with high missingness or low event counts, the final model consisted of 15 variables, including demographic characteristics, clinical factors, sites of metastasis, and treatment modalities.

To complement traditional regression approaches and explore potential nonlinear associations, we developed random survival forest (RSF) models using the randomForestSRC R package.^23,24^ Model discrimination was evaluated using 10-fold cross-validation and was quantified by the concordance index (C-index)^25^ with mean values and 95% CIs reported. To enhance interpretability, SHAP were applied to quantify the association of each demographic, clinical, and socioeconomic variable with survival.^26^ SHAP values were computed using the shap Python library,^26^ while data preprocessing and visualizations were conducted using pandas^27^ and matplotlib^28^ in Python.

## Results

From 2010 to 2021, a total of 499 670 NSCLC cases were identified in the SEER database, 18 595 of which involved patients aged 18 to 50 years (eFigure 1 in Supplement 1). The mean (SD) age at diagnosis was 44.6 (6.0) years, with 15 597 patients (83.9%) aged 40 to 50 years. A total of 9710 patients (52.2%) were female and 8885 (47.8%) were male. The racial and ethnic distribution was as follows: 2338 patients (12.6%) were Hispanic, 100 (0.5%) were American Indian or Alaska Native, 2229 (12.0%) were non-Hispanic Asian or Pacific Islander, 2693 (14.5%) were non-Hispanic Black, 11 162 (60.0%) were non-Hispanic White, and 73 (0.4%) were of unknown race and ethnicity. Among these patients, 15 854 (85.3%) resided in urban areas, and 6651 (35.8%) had household incomes between $55 000 and $75 000. At diagnosis, 4369 patients (23.5%) had bone metastases and 3659 (19.7%) had brain metastases. Most patients received chemotherapy (10 706 [57.6%]) or radiation therapy (7670 [41.2%]), whereas only 1354 (7.3%) underwent surgical treatment (eTable 1 in Supplement 1).

**Figure 1.**
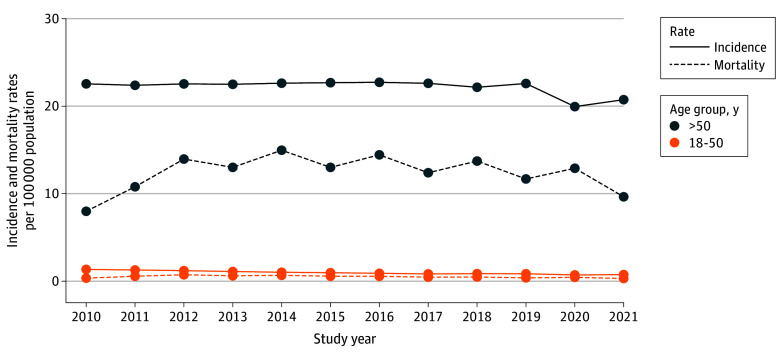
Age-Specific Annual Incidence and Mortality Rates Among Patients With Non–Small Cell Lung Cancer Data were obtained from 2010 to 2021. Incidence and mortality rates were measured per 100 000 population.

While the incidence and mortality of NSCLC declined in patients older than 50 years, rates remained stable among younger adults, resulting in a relative increase in the proportion of early-onset cases. Incidence and mortality rates per 100 000 were consistently higher in individuals older than 50 years compared with those aged 18 to 50 years, with a decline after 2019 in lung cancer incidence among older patients (Figure 1). More than half of the patients aged 18 to 50 years (9929 [53.4%]) were diagnosed with stage IV disease, and adenocarcinoma was the most common histological subtype (10 965 [59.0%]). Stage distribution varied across histological subtypes and age groups; stage IV was most common in adenocarcinoma and unspecified histology, whereas large cell carcinoma was more frequently diagnosed at earlier stages (eFigure 2 in Supplement 1).

### Survival Analysis by Demographic and Clinical Subgroups

**Figure 2.**
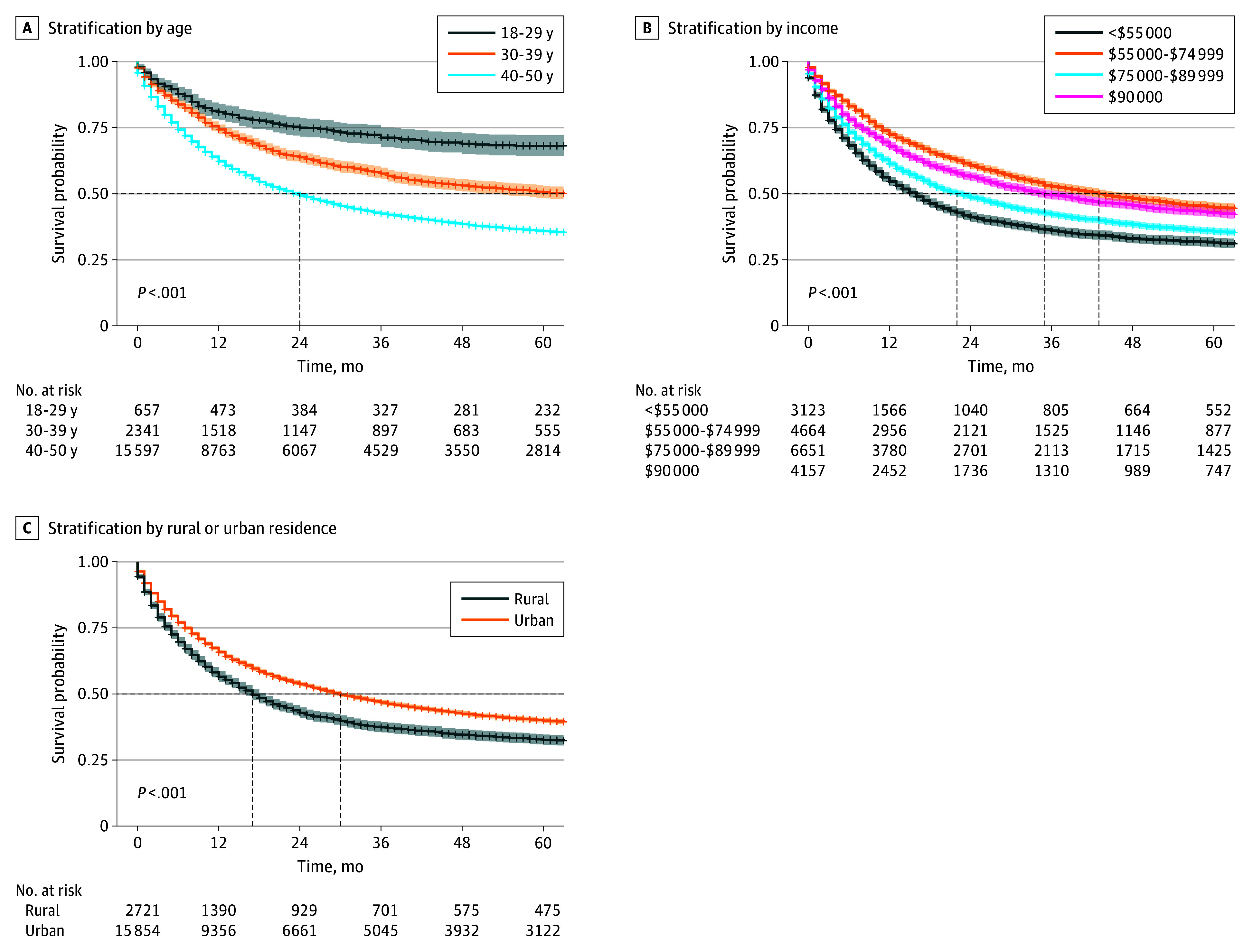
Kaplan-Meier Survival Curves for Patients With Non–Small Cell Lung Cancer Shaded areas indicate 95% CIs. Dashed horizontal lines indicate the 50% survival probability; dashed vertical lines indicate the median survival time for each subgroup.

The mean (SD) OS was 31.6 (36.4) months, and the median OS was 16 (IQR, 5-43) months. Subgroup analysis showed OS varied by age, sex, household income, and stage (Figure 2 and eFigures 3-5 in Supplement 1). Patients aged 18 to 29 years had the longest median survival at 34 (IQR, 9-77) months, compared with 15 (IQR, 5-43) months among those aged 40 to 50 years. Female patients lived longer than male patients (median, 20 [IQR, 6-54] vs 12 [IQR, 4-36] months). Higher household income was associated with better survival, with a median of 20 (IQR, 7-47) months in the highest income group. Patients with stage I disease had the longest median survival (58 [IQR, 27-97] months), and those with stage IV disease had the shortest (9 [IQR, 3-23] months) (Figure 2).

### Explainable Models Associated With Survival 

In multivariable Cox proportional hazards regression analysis, older age, male sex, single marital status, rural residence, and lower household income were independently associated with poorer OS (Table). Patients aged 40 to 50 years had a higher mortality risk compared with those aged 18 to 29 years (HR, 1.86; 95% CI, 1.57-2.20), and male patients had worse outcomes than female patients (HR, 1.19; 95% CI, 1.14-1.25). Compared with patients with a household income greater than $90 000, those earning less than $55 000 had a significantly increased risk of death (HR, 1.45; 95% CI, 1.33-1.58).

**Table.** Cox Proportional Hazards Regression Analysis of Demographic and Clinical Factors in the Study Cohort^a^

Factor	Univariable analysis (n = 18 595)			Multivariable analysis (n = 15 023)		
	Event, No./total No. (%)	HR (95% CI)	*P* value	Event, No./total No. (%)	HR (95% CI)	*P* value
Age, y						
18-29	185/657 (28.2)	1 [Reference]	NA	139/530 (26.2)	1 [Reference]	NA
30-39	1004/2341 (42.9)	1.74 (1.48-2.03)	<.001	802/1920 (41.8)	1.36 (1.13-1.63)	<.001
40-50	8931/15 597 (57.3)	2.66 (2.30-3.07)	<.001	7062/12 573 (56.2)	1.86 (1.57-2.20)	<.001
Sex						
Female	4779/9710 (49.2)	1 [Reference]	NA	3848/8053 (47.8)	1 [Reference]	NA
Male	5341/8885 (60.1)	1.46 (1.40-1.51)	<.001	4155/6970 (59.6)	1.19 (1.14-1.25)	<.001
Marital status						
Married	4678/9113 (51.3)	1 [Reference]	NA	3894/7753 (50.2)	1 [Reference]	NA
Single	4911/8417 (58.3)	1.31 (1.26-1.37)	<.001	3733/6534 (57.1)	1.28 (1.22-1.34)	<.001
Unknown or other	531/1065 (49.9)	1.04 (0.95-1.14)	.37	376/736 (51.1)	1.14 (1.02-1.26)	.02
Race						
Hispanic	1155/2338(49.4)	0.90 (0.84-0.95)	<.001	867/1789 (48.5)	0.87 (0.81-0.94)	<.001
Non-Hispanic American Indian or Alaska Native	63/100 (63.0)	1.15 (0.90-1.48)	.26	34/58 (58.6)	1.10 (0.78-1.54)	.58
Non-Hispanic Asian or Pacific Islander	1112/2229 (49.9)	0.87 (0.81-0.92)	<.001	880/1830 (48.1)	0.76 (0.71-0.82)	<.001
Non-Hispanic Black	1615/2693 (60.0)	1.24 (1.17-1.31)	<.001	1221/2062 (59.2)	1.04 (0.98-1.11)	.21
Non-Hispanic White	6161/11 162 (55.2)	1 [Reference]	NA	4989/9237 (54.0)	1 [Reference]	NA
Unknown	14/73 (19.2)	0.36 (0.21-0.60)	<.001	12/47 (25.5)	0.57 (0.32-1.00)	.052
Residence						
Rural	1661/2721 (61.0)	1.28 (1.22-1.35)	<.001	1331/2168 (61.4)	1.08 (1.00-1.16)	.052
Urban	8447/15 854 (53.3)	1 [Reference]	NA	6672/12 855 (51.9)	1 [Reference]	NA
Household income						
<$55 000	1973/3123 (63.2)	1.63 (1.53-1.73)	<.001	1560/2475 (63.0)	1.45 (1.33-1.58)	<.001
$55 000-$74 999	3951/6651 (59.4)	1.37 (1.30-1.44)	<.001	3037/5218 (58.2)	1.30 (1.23-1.39)	<.001
$75 000-$89 999	2032/4157 (48.9)	1.12 (1.050-1.19)	<.001	1622/3375 (48.1)	1.09 (1.02-1.17)	.01
≥$90 000	2164/4664 (46.4)	1 [Reference]	NA	1784/3955 (45.1)	1 [Reference]	NA
Time to treatment, mean (SD), mo	0.98 (1.26)	0.87 (0.85-0.88)	<.001	0.98 (1.26)	0.91 (0.89-0.93)	<.001
Overall stage						
I	325/3257 (10.0)	1 [Reference]	NA	285/3056 (9.3)	1 [Reference]	NA
II	318/1175 (27.1)	2.95 (2.53-3.45)	<.001	286/1092 (26.2)	3.14 (2.66-3.71)	<.001
III	1624/3046 (53.3)	7.87 (6.99-8.87)	<.001	1416/2681 (52.8)	8.08 (7.03-9.30)	<.001
IV	7336/9929 (73.9)	17.15 (15.34-19.19)	<.001	6016/8194 (73.4)	17.47 (15.28-19.96)	<.001
Histological subtype						
Adenocarcinoma	6440/10 965 (58.7)	1 [Reference]	NA	5402/9431 (57.3)	1 [Reference]	NA
Large cell carcinoma	1409/3696 (38.1)	0.56 (0.52-0.59)	<.001	1035/2979 (34.7)	0.92 (0.86-0.99)	.02
Other or unspecified	732/1624 (45.1)	0.90 (0.84-0.98)	.009	297/701 (42.4)	1.23 (1.10-1.39)	<.001
Squamous cell carcinoma	1539/2310 (66.6)	1.30 (1.23-1.38)	<.001	1269/1912 (66.4)	1.42 (1.33-1.51)	<.001
Bone metastasis						
No	6776/14 226 (47.6)	1 [Reference]	NA	5245/11 397 (46.0)	1 [Reference]	NA
Yes	3344/4369 (76.5)	2.68 (2.57-2.80)	<.001	2758/3626 (76.1)	1.30 (1.23-1.38)	<.001
Brain metastasis						
No	7363/14 936 (49.3)	1 [Reference]	NA	5611/11 827 (47.4)	1 [Reference]	NA
Yes	2757/3659 (75.3)	2.27 (2.17-2.38)	<.001	2392/3196 (74.8)	1.06 (0.996-1.12)	.07
Liver metastasis						
No	8585/16 681 (51.5)	1 [Reference]	NA	6814/13 524 (50.4)	1 [Reference]	NA
Yes	1535/1914 (80.2)	2.77 (2.63-2.93)	<.001	1189/1499 (79.3)	1.45 (1.35-1.54)	<.001
Surgery						
No or unknown	9194/17 241 (53.3)	1 [Reference]	NA	7117/13 728 (51.8)	1 [Reference]	NA
Yes	926/1354 (68.4)	1.40 (1.34-1.53)	<.001	886/1295 (68.4)	0.80 (0.74-0.86)	<.001
Radiation therapy						
No or unknown	4780/10 925 (43.8)	1 [Reference]	NA	2970/7825 (38.0)	1 [Reference]	NA
Yes	5340/7670 (69.6)	1.95 (1.82-2.03)	<.001	5033/7198 (69.9)	1.27 (1.20-1.34)	<.001
Chemotherapy						
No or unknown	3190/7889 (40.4)	1 [Reference]	NA	1577/5145 (30.6)	1 [Reference]	NA
Yes	6930/10 706 (64.7)	1.68 (1.61-1.76)	<.001	6426/9878 (65.0)	0.68 (0.64-0.73)	<.001

Abbreviations: HR, hazard ratio; NA, not applicable.

^a^ Includes 18 595 patients diagnosed from 2010 to 2021. Each event represents a death attributable to non–small cell lung cancer.

Stage IV disease was the strongest factor associated with mortality (HR, 17.47; 95% CI, 15.28-19.96). Bone (HR, 1.30; 95% CI, 1.23-1.38) and liver metastases (HR, 1.45; 95% CI, 1.35-1.54) were associated with increased risk, while brain metastases were not associated with a significantly increased risk. Squamous cell carcinoma (HR, 1.42; 95% CI, 1.33-1.51) and other or unspecified histological subtypes (HR, 1.23; 95% CI, 1.10-1.39) were linked to poorer survival compared with adenocarcinoma. Patients who received chemotherapy (HR, 0.68; 95% CI, 0.64-0.73) or underwent surgery (HR, 0.80; 95% CI, 0.74-0.86) had lower mortality. Full univariable and multivariable model results are presented in the Table.

**Figure 3.**
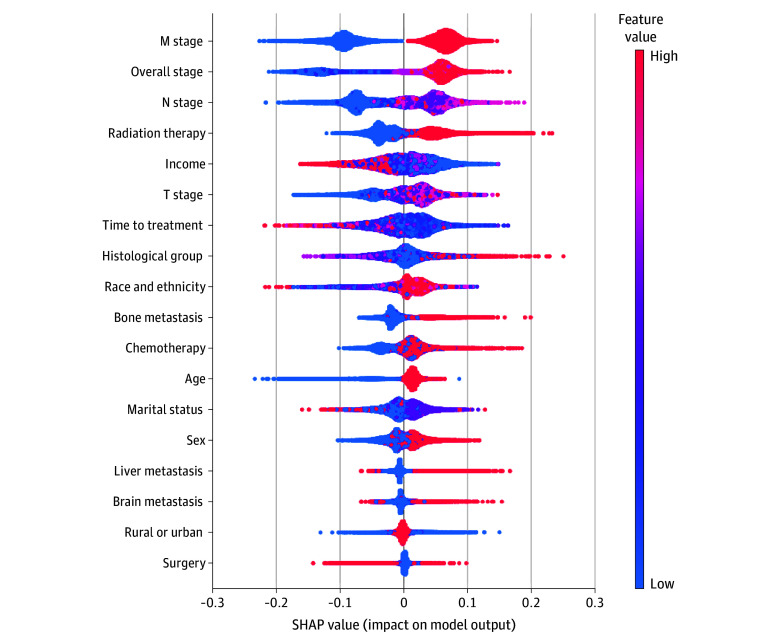
Input Variable Contributions to the Survival Model’s Risk Estimates SHAP indicates Shapley additive explanations.

To complement the Cox proportional hazards regression analysis, which assumes linear effects of covariates on the log-hazard, an RSF model was developed to capture nonlinear associations and assess their performance. The Cox proportional hazards model demonstrated slightly higher discrimination (C-index, 0.774; 95% CI, 0.769-0.779) compared with the RSF model (0.765; 95% CI, 0.759-0.771), although both showed comparable accuracy. While the Cox proportional hazards model provided interpretable HRs, the RSF model captured complex interactions. SHAP-enhanced feature importance analysis identified overall stage, radiation therapy, household income, and time from diagnosis to treatment initiation as the most influential factors associated with survival in both models (Figure 3 and eFigure 6 in Supplement 1).

### Survival Differences by Time to Treatment and Sociodemographic Factors Across Disease Stages

**Figure 4.**
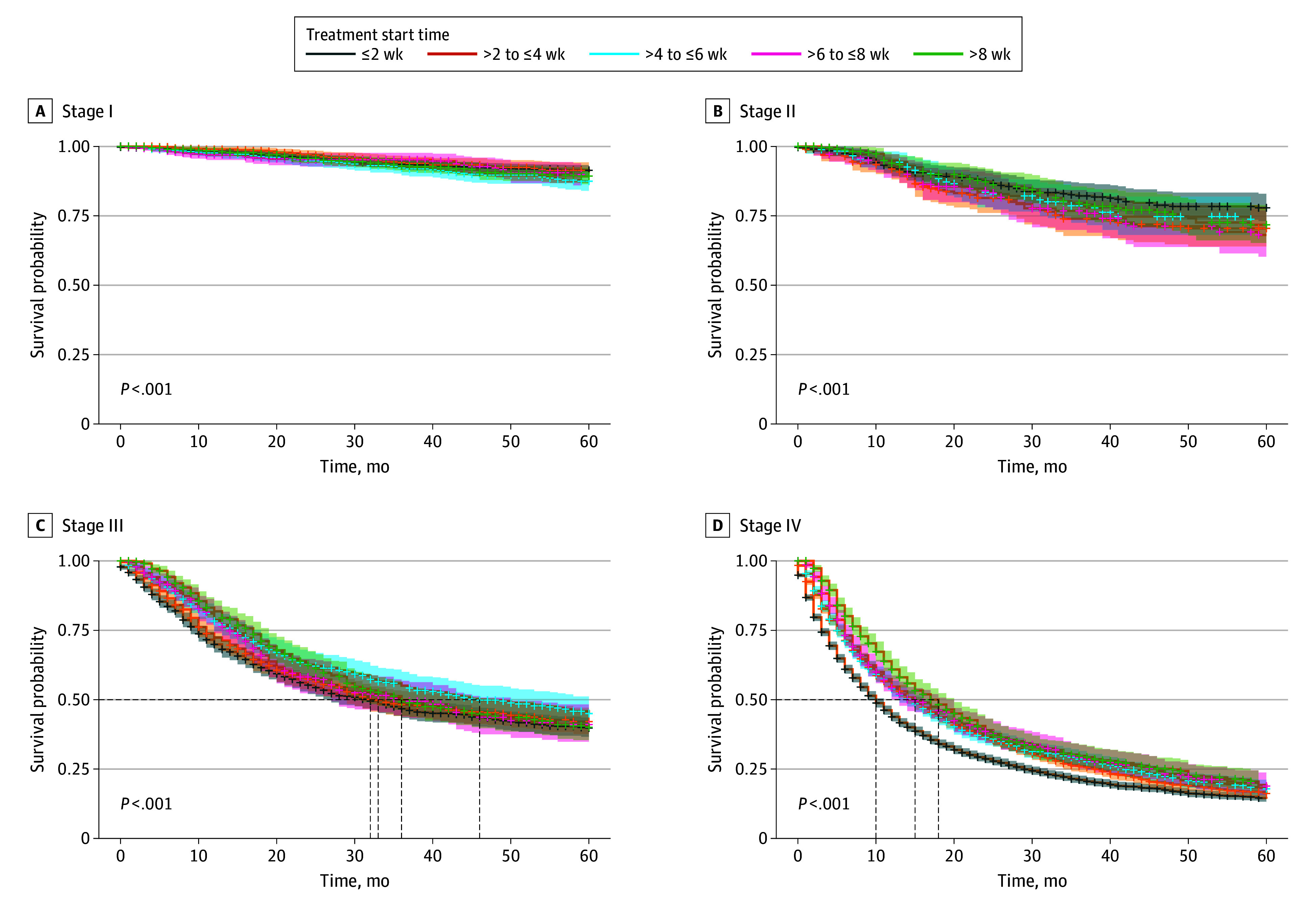
Kaplan-Meier Curves of Survival by Diagnosis to Treatment Time and Stage A, Hazard ratios (HRs) were 1.06 (95% CI, 0.74-1.51) for greater than 2 to 4 weeks; 1.53 (95% CI, 1.10-2.12) for greater than 4 to 6 weeks; 1.26 (95% CI, 0.84-1.90) for greater than 6 to 8 weeks; and 1.30 (95% CI, 0.94-1.80) for greater than 8 weeks. B, HRs were 1.30 (95% CI, 0.93-1.83) for greater than 2 to 4 weeks; 1.16 (95% CI, 0.81-1.66) for greater than 4 to 6 weeks; 1.47 (95% CI, 1.02-2.10) for greater than 6 to 8 weeks; and 1.04 (95% CI, 0.73-1.48) for greater than 8 weeks. C, HRs were 0.95 (95% CI, 0.82-1.10) for greater than 2 to 4 weeks; 0.83 (95% CI, 0.71-0.96) for greater than 4 to 6 weeks; 0.89 (95% CI, 0.74-1.08) for greater than 6 to 8 weeks; and 0.88 (95% CI, 0.75-1.03) for greater than 8 weeks. D, HRs were 0.81 (95% CI, 0.76-0.86) for greater than 2 to 4 weeks; 0.79 (95% CI, 0.73-0.85) for greater than 4 to 6 weeks; 0.75 (95% CI, 0.67-0.83) for greater than 6 to 8 weeks; and 0.69 (95% CI, 0.63-0.76) for greater than 8 weeks. Shaded areas indicate 95% CIs. Dashed horizontal lines indicate the 50% survival probability; dashed vertical lines indicate the median survival time for each subgroup.

Mortality events by diagnosis-to-treatment time and cancer stage are presented in eTable 2 in Supplement 1. Importantly, across stage-stratified models, diagnosis-to-treatment time was associated with survival (Figure 4). In stage I disease, early treatment within 2 weeks was associated with better survival, while starting treatment between more than 4 and 6 weeks showed worse outcomes (HR, 1.53; 95% CI, 1.10-2.12; *P* = .01). In contrast, for stage IV cancer, early systemic treatment within 2 weeks was linked to significantly worse survival, with those treated after 8 weeks showing improved outcomes (HR, 0.69; 95% CI, 0.63-0.76; *P* < .001).

Sociodemographic disparities in survival were also observed across different stages (eFigures 7 and 8 in Supplement 1). Notably, the adverse association of rural residence and low household income (<$55 000) with OS was more pronounced in early-stage (stages I and II) NSCLC compared with advanced-stage (stages III and IV) disease, with HRs for rural residency of 1.65 (95% CI, 1.25-2.18) for stage 1 and 1.60 (95% CI, 1.23-2.08) for stage II vs 1.31 (95% CI, 1.15-1.48) for stage III and 1.37 (95% CI, 1.28-1.46) for stage IV, and HRs for low household income of 1.96 (95% CI, 1.52-2.55) for stage I and 1.63 (95% CI, 1.27-2.09) for stage II vs 1.27 (95% CI, 1.13-1.43) for stage III and 1.46 (95% CI, 1.38-1.55) for stage IV (eFigures 7 and 8 in Supplement 1).

### Sensitivity Analysis

A sensitivity analysis excluding patients with unknown race and ethnicity (n = 73) yielded consistent results. Race remained associated with survival, with Hispanic and non-Hispanic Asian or Pacific Islander patients continuing to show improved survival compared with non-Hispanic White patients.

To examine potential diagnostic bias due to the COVID-19 pandemic, we conducted a sensitivity analysis excluding patients diagnosed in 2020 and 2021. The results were consistent with the primary analysis, reinforcing the robustness of key factors such as race and ethnicity, household income, rural vs urban status, and time to treatment.

## Discussion

Our analysis revealed a significant decline in NSCLC incidence and mortality among older adults, whereas the incidence of early-onset cases remained stable—an emerging pattern that may indicate a potential rise in mortality among patients younger than 50 years. Early-onset NSCLC cancer was more prevalent in female patients, yet OS was significantly poorer in male patients, suggesting potential differences in disease biology between sexes and highlighting factors that may influence treatment continuation. Additionally, we identified stage IV disease and metastases as key factors associated with poor survival in patients with early-onset NSCLC, consistent across both Cox proportional hazards regression and SHAP-enhanced models. SHAP values were used to interpret model estimations due to their ability to provide both local (individual-level) and global (population-level) explanations, to handle correlated features more robustly than permutation-based methods, and to ensure consistent, theoretically grounded attribution through game theory.^26^ These properties are particularly valuable for clinical applications, where interpretability at multiple levels is essential. In addition to clinical variables, SHAP analysis highlighted sociodemographic factors, such as rural residence and household income, as important contributors to survival outcomes. By combining traditional and machine learning approaches, we captured both interpretable hazard estimates and complex variable interactions. The consistency of results across methods supports the robustness of our findings and highlights the utility of explainable artificial intelligence tools in oncologic research. Importantly, in machine learning models, modifiable factors such as stage at diagnosis, time to treatment initiation, household income, and residential setting demonstrated greater significance than nonmodifiable variables such as histology, age, and race and ethnicity.

More than half of patients presented with advanced-stage disease, consistent with the findings of prior studies.^5,29^ This likely reflects delayed symptom recognition, limited health care access, and the misattribution of early signs to benign conditions, all of which reinforce the need for improved early detection in this age group. The current eligibility criteria for lung cancer screening underestimate the potential impact of lung cancer screening in this age group, as adults younger than 50 years were not included. As a result, early-stage cancers are often missed, limiting curative-intent treatment options and worsening long-term outcomes. Our findings support the development of alternative symptom-driven or risk-adapted screening strategies for selecting young adults at high risk for developing lung cancer. Giangregorio et al^30^ indicated that analysis of cough sounds using artificial intelligence, combined with smoking history, could differentiate patients with lung cancer from healthy individuals. Currently, an ongoing trial^31^ is studying the diagnosis rate of lung cancer with mobile screening using low-dose computed tomography of the chest in individuals aged 40 to 54 years who have a smoking history of 30 pack-years and who are either current smokers or have quit within the last 15 years. Another ongoing study, the EQUAL (*EGFR* ctDNA Quantitative Assessment for Lung Cancer) trial,^32^ aims to detect *EGFR* mutations using blood-circulating cell-free DNA in patients who never smoked and are of Asian or Hispanic-Latinx descent aged 50 to 80 years. The trial will enroll participants aged 40 to 49 years if they also have a family history of *EGFR*-mutant lung cancer or other risk factors other than tobacco. Additionally, educational interventions are required to improve symptom awareness and prompt evaluation. Studies such as the one by Saab et al^33^ show important gaps in patient awareness and disclosure related to lung cancer symptoms, which may be due in part to a sense of immortality or embarrassment regarding medical conditions that is often seen in this age group. Collectively, these efforts may help close the diagnostic gap and improve survival outcomes in this underserved and often overlooked population.

Socioeconomic disparities were especially evident in early-stage disease, where patients with lower household income and rural residence had significantly worse survival. This may reflect reduced access to timely surgical care or follow-up. Prior studies have linked low SES to delayed or missed adjuvant therapy^34^ and racial disparities in curative treatment^35^ despite similar incidence rates. Di Vanna et al^36^ demonstrated a consistent impact of lower income on cancer survival, including lung cancer, by analyzing SEER database data. Income is also associated with insurance status, which in turn is associated with stage at diagnosis^37^ and OS^38^ and could be explored in future models. Our findings add evidence that emphasizes the need for policies that reduce structural barriers to surgery and radiation therapy and ensure equitable access to care.

While delayed diagnosis and consequent advanced stage at presentation are among the most critical prognostic factors in adults 50 years or younger with NSCLC, our study is the first, to our knowledge, to assess the association of delayed start of treatment initiation after diagnosis with survival at a population level in this patient group. Our findings suggest that time from diagnosis to treatment initiation was associated with OS. In stage I or II disease, initiating treatment within 2 weeks was associated with improved survival, likely due to more straightforward and single-modality treatment such as surgery or targeted radiation therapy. In contrast, prompt treatment (within 2 weeks) in stage IV disease was associated with to markedly poorer survival, possibly reflecting urgency in the setting of high tumor volume, symptomatic disease, and rapidly progressing disease. For stage III or IV disease, slightly longer intervals may be appropriate to allow for molecular profiling and comprehensive treatment planning, including radiation simulation. Our findings are consistent with a National Cancer Database analysis^39^ showing that among patients with metastatic cancer, initiating treatment between 4.0 and 6.0 weeks after diagnosis was associated with a lower risk of death compared with treatment within 1 to 4 weeks (HR, 0.75; 95% CI 0.74–0.76). In contrast, delaying surgery beyond 6 weeks was associated with an increased risk of death (HR, 1.17; 95% CI 1.14-1.20).^39^ These results support the need for individualized treatment timelines tailored to disease stage and highlight the importance of comprehensive workups, including genomic testing and immune marker assessment, in advanced-stage NSCLC to enable more personalized and durable treatment strategies.

### Strengths and Limitations

This study is strengthened by its use of a large and nationally representative dataset and the integration of both statistical and explainable machine learning methods. However, it also has several limitations. Socioeconomic variables, including household income, were derived from zip code–level data and thus do not reflect individual-level socioeconomic status. Race and ethnicity were categorized using aggregated SEER-defined classifications, which grouped Asian individuals with Native Hawaiian and Other Pacific Islander individuals. This may mask important subgroup-level differences within these 2 populations and should be explored in future studies. Smoking status was not included due to limited availability across the dataset and linked sources.

Although we adjusted for a wide range of demographic, clinical, and area-level variables, unmeasured factors such as access to care, physician-level variation, or health care infrastructure may still influence survival outcomes. From the clinical perspective, genomic alterations and tumor immune markers play a critical role in guiding lung cancer treatment, especially among adults who are more likely to be nonsmokers and carry targetable mutations. However, molecular data were not available in the SEER database at the time of this analysis.

Finally, although we used 10-fold cross-validation to reduce overfitting and mitigate outcome imbalance, the lack of external validation remains a limitation. Future research should assess the generalizability of our findings in independent cohorts.

## Conclusions

In this population-based cohort study, survival among adults aged 18 to 50 years with early-onset NSCLC was shaped by both clinical and modifiable sociodemographic factors. Advanced stage at diagnosis remained the most prominent factor associated with poor outcome, but disparities in household income, rural residence, and treatment timing were also significant factors in early-stage survival. These findings call for the development of risk-adapted screening strategies, improved access to curative treatments, and policy interventions to address socioeconomic barriers in this underserved population.

## Notes

Supplement 1. **eFigure 1.** Patient Selection CONSORT Flowchart **eTable 1.** Baseline Characteristics of Study Populations for Diagnosis 2010-2021 **eFigure 2.** NSCLC Stage Distribution by Histological Subtype and Age Group **eFigure 3.** Kaplan-Meier Survival Curves by Sex **eFigure 4.** Kaplan-Meier Survival Curves by Race **eFigure 5.** Kaplan-Meier Survival Curves by Histological Subtypes **eFigure 6.** Global Feature Importance Ranked by Mean Absolute SHAP Values From the Random Survival Forest Model **eTable 2.** Number of Patients and Events by Treatment Group and Overall Stage **eFigure 7.** Kaplan-Meier Survival by Rural vs Urban Residence Across Stages I-IV **eFigure 8.** Kaplan-Meier Survival by Income Group Across Stages I-IV

Supplement 2.
Data Sharing Statement
