# Supplementary material for: Survival Determinants and Sociodemographic Disparities in Early-Onset Non–Small Cell Lung Cancer
Source: JAMA Netw Open. 2025 Oct 13;8(10):e2537307. doi: 10.1001/jamanetworkopen.2025.37307 (PMC12519311; doi:10.1001/jamanetworkopen.2025.37307)
Supplement: Supplement 1. — eFigure 1. Patient Selection CONSORT Flowchart eTable 1. Baseline Characteristics of Study Populations for Diagnosis 2010-2021 eFigure 2. NSCLC Stage Distribution by Histological Subtype and Age Group eFigure 3. Kaplan-Meier Survival Curves by Sex eFigure 4. Kaplan-Meier Survival Curves by Race eFigure 5. Kaplan-Meier Survival Curves by Histological Subtypes eFigure 6. Global Feature Importance Ranked by Mean Absolute SHAP Values From the Random Survival Forest Model eTable 2. Number of Patients and Events by Treatment Group and Overall Stage eFigure 7. Kaplan-Meier Survival by Rural vs Urban Residence Across Stages I-IV eFigure 8. Kaplan-Meier Survival by Income Group Across Stages I-IV [file jamanetwopen-e2537307-s001.pdf]

Supplementary Online Content

Kar İ, Vhora F, Bou Zerdan M, et al. Survival determinants and sociodemographic disparities in early-onset non–small cell lung cancer. *JAMA Netw Open.* 2025;8(10):e2537307. doi:10.1001/jamanetworkopen.2025.37307

eFigure 1. Patient Selection CONSORT Flowchart

eTable 1. Baseline Characteristics of Study Populations for Diagnosis 2010-2021

eFigure 2. NSCLC Stage Distribution by Histological Subtype and Age Group

eFigure 3. Kaplan-Meier Survival Curves by Sex

eFigure 4. Kaplan-Meier Survival Curves by Race

eFigure 5. Kaplan-Meier Survival Curves by Histological Subtypes

eFigure 6. Global Feature Importance Ranked by Mean Absolute SHAP Values From the Random Survival Forest Model

eTable 2. Number of Patients and Events by Treatment Group and Overall Stage

eFigure 7. Kaplan-Meier Survival by Rural vs Urban Residence Across Stages I-IV

eFigure 8. Kaplan-Meier Survival by Income Group Across Stages I-IV

This supplementary material has been provided by the authors to give readers additional information about their work.

© 2025 Kar İ et al. *JAMA Network Open*.

eFigure 1. Patient Selection CONSORT Flowchart

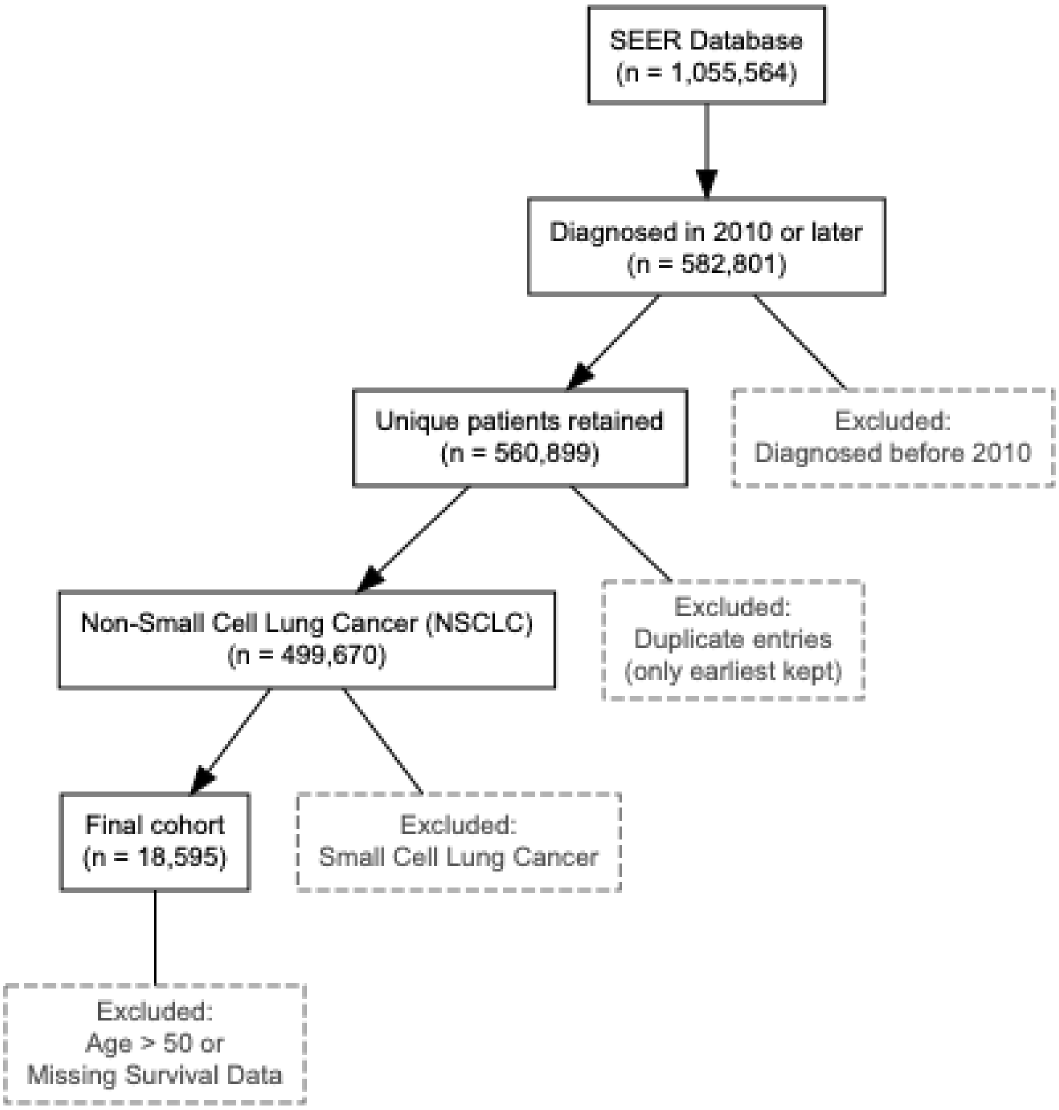

© 2025 Kar i et al. *JAMA Network Open*.

eTable 1. Baseline Characteristics of Study Populations for Diagnosis 2010-2021

Characteristic	2010-2021 (n = 18,595)	Survival Time	
**Age, years**	**No. (%)**	**Mean (SD)**	**Median (Q1-Q3)**
18-29	657 (3.5)	48.04 (43.16)	34 (9-77)
30-39	2,341 (12.6)	36.98 (37.93)	22 (7-56)
40-50	15,597 (83.9)	30.14 (35.56)	15 (5-43)
**Sex**			
Female	9,710 (52.2)	35.71 (37.83)	20 (6-54)
Male	8,885 (47.8)	27.17 (34.13)	12 (4-36)
**Marital Status**			
Married	9,113 (49.0)	34.6 (37.26)	20 (6-51)
Single	8,417 (45.3)	28.22 (34.78)	13 (4-39)
Unknown/Other	1,065 (5.7)	33.21 (38.47)	16 (4-50)
**Race**			
Hispanic	2,338 (12.6)	30.76 (34.7)	18 (5-42)
Non-Hispanic AI/AN	100 (0.5)	33.59 (39.92)	17 (4-51.25)
Non-Hispanic Asian/Pacific Islander	2,229 (12.0)	31.34 (32.36)	19 (7-44)
Non-Hispanic Black	2,693 (14.5)	26.38 (34)	12 (4-35)
Non-Hispanic White	11,162 (60.0)	33.12 (37.83)	16 (5-50)
Unknown	73 (0.4)	32.34 (36.21)	16 (3-53)
**Rural/Urban**			
Rural	2,721 (14.6)	27.93 (35.4)	12 (3-38)
Urban	15,854 (85.3)	32.27 (36.49)	17 (5-47)
NA	20 (0.1)	29.75 (35.64)	9 (3.75-55.5)
**Median household income (2022 adj.)**			
< $55,000	3,123 (16.8)	27.78 (35.7)	12 (3-37)
$55,000 - $75,000	6,651 (35.8)	32.74 (38.1)	14 (5-49)
$75,000 - $89,999	4,157 (22.4)	31.18 (35.23)	17 (5-45)
> $90,000	4,664 (25.1)	33.02 (35.06)	20 (7-47)
**Overall Stage**			
1	3,257 (17.5)	62.74 (41.38)	58 (27-97)
2	1,175 (6.3)	55.67 (42.26)	43 (18-89.5)
3	3,046 (16.4)	34.71 (35.57)	20 (8-50)
4	9,929 (53.4)	17.78 (23.78)	9 (3-23)
NA	1,188 (6.4)	30.43 (37.66)	13 (3-45)
**Histology Group**			
Adenocarcinoma	10,965 (59.0)	30.28 (34.38)	15 (5-43)
Large Cell Carcinoma	3,696 (19.9)	42.6 (42.32)	26 (7-71)
Other/Unspecified	1,624 (8.7)	25.51 (34.36)	10 (2-34)
Squamous Cell Carcinoma	2,310 (12.4)	24.78 (32.64)	11 (4-29)
**Mets at DX (2010)**			
**Bone (Yes)**	4,369 (23.5)	14.61 (19.76)	7 (2-19)
**Brain (Yes)**	3,659 (19.7)	16.55 (21.9)	8 (3-21)

© 2025 Kar i et al. *JAMA Network Open*.

**Liver (Yes)**	1,914 (10.3)	12.27 (18.11)	6 (2-15)
**Treatment**			
**Surgery (Yes)**	1,354 (7.3)	25.2 (31.23)	11 (4-33)
**Chemotherapy (Yes)**	10,706 (57.6)	27.12 (30.48)	16 (6-36)
**Radiation (Yes)**	7,670 (41.2)	23.48 (28.66)	12 (5-30)
**Event (Death)**	10,120 (54.4)	14.92 (18.18)	9 (3-20)

© 2025 Kar İ et al. *JAMA Network Open*.

eFigure 2. NSCLC Stage Distribution by Histological Subtype and Age Group

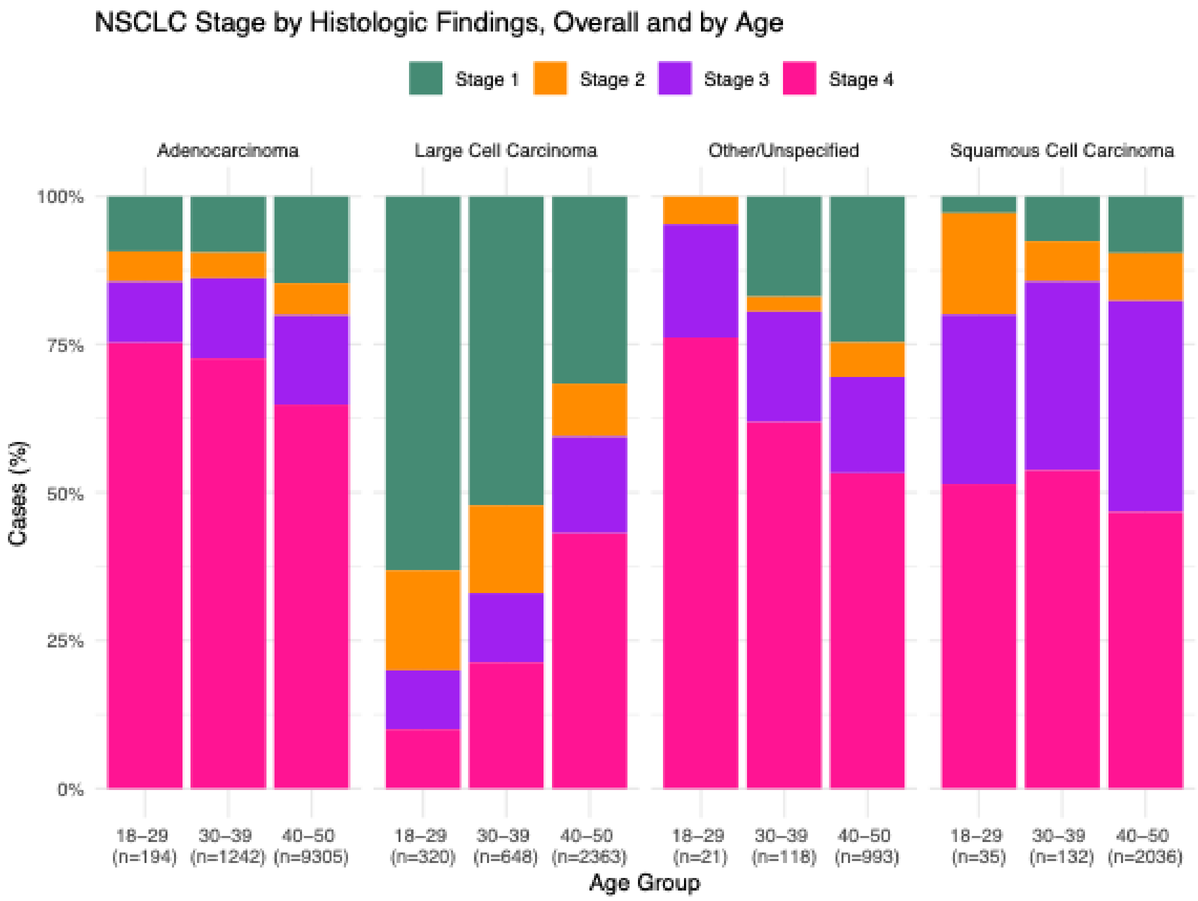

© 2025 Kar i et al. *JAMA Network Open*.

eFigure 3. Kaplan-Meier Survival Curves by Sex

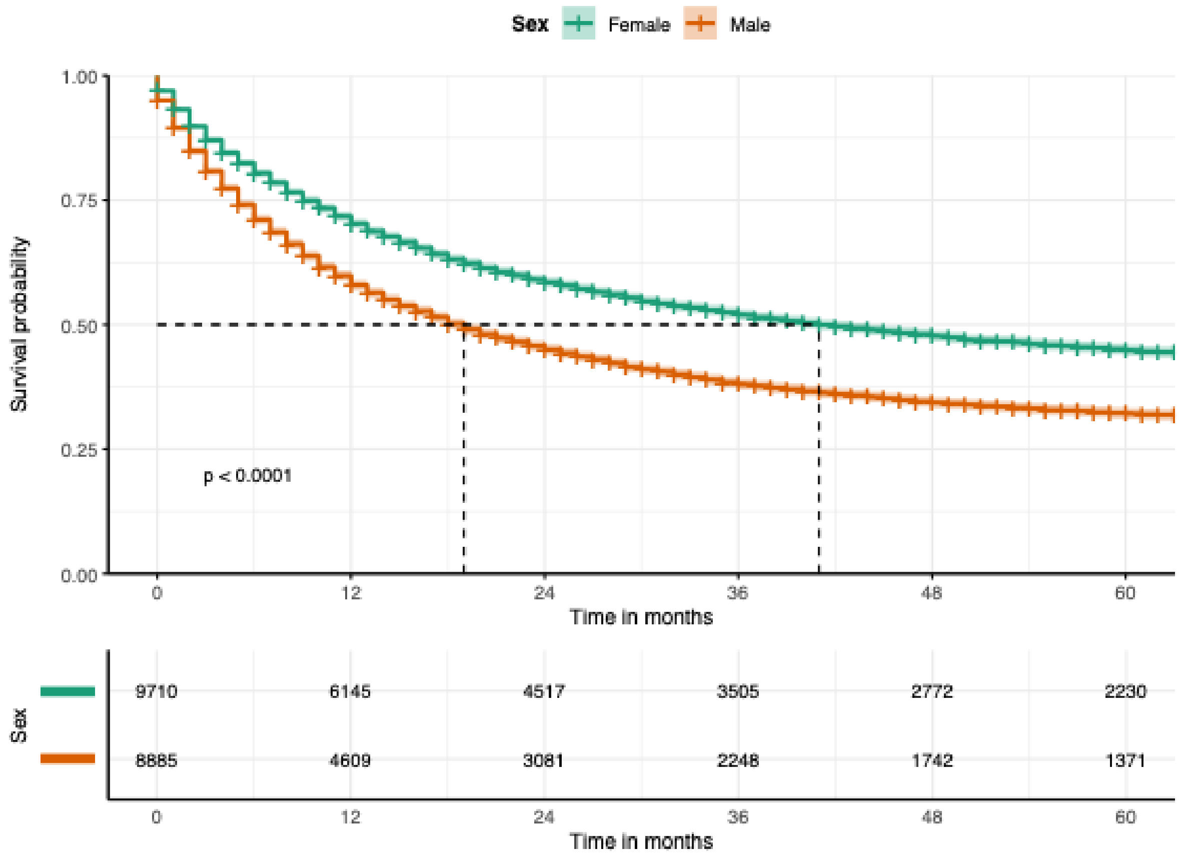

© 2025 Kar i et al. *JAMA Network Open*.

eFigure 4. Kaplan-Meier Survival Curves by Race

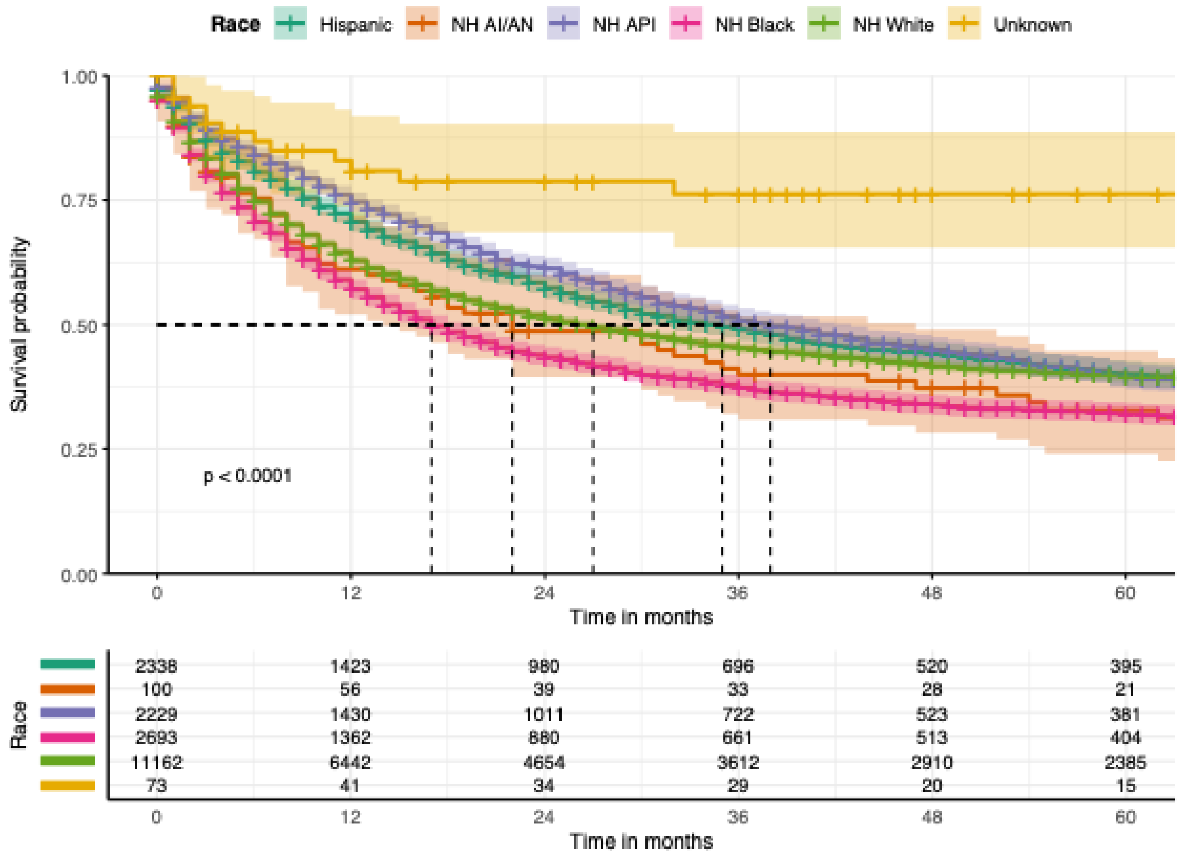

© 2025 Kar i et al. *JAMA Network Open*.

eFigure 5. Kaplan-Meier Survival Curves by Histological Subtypes

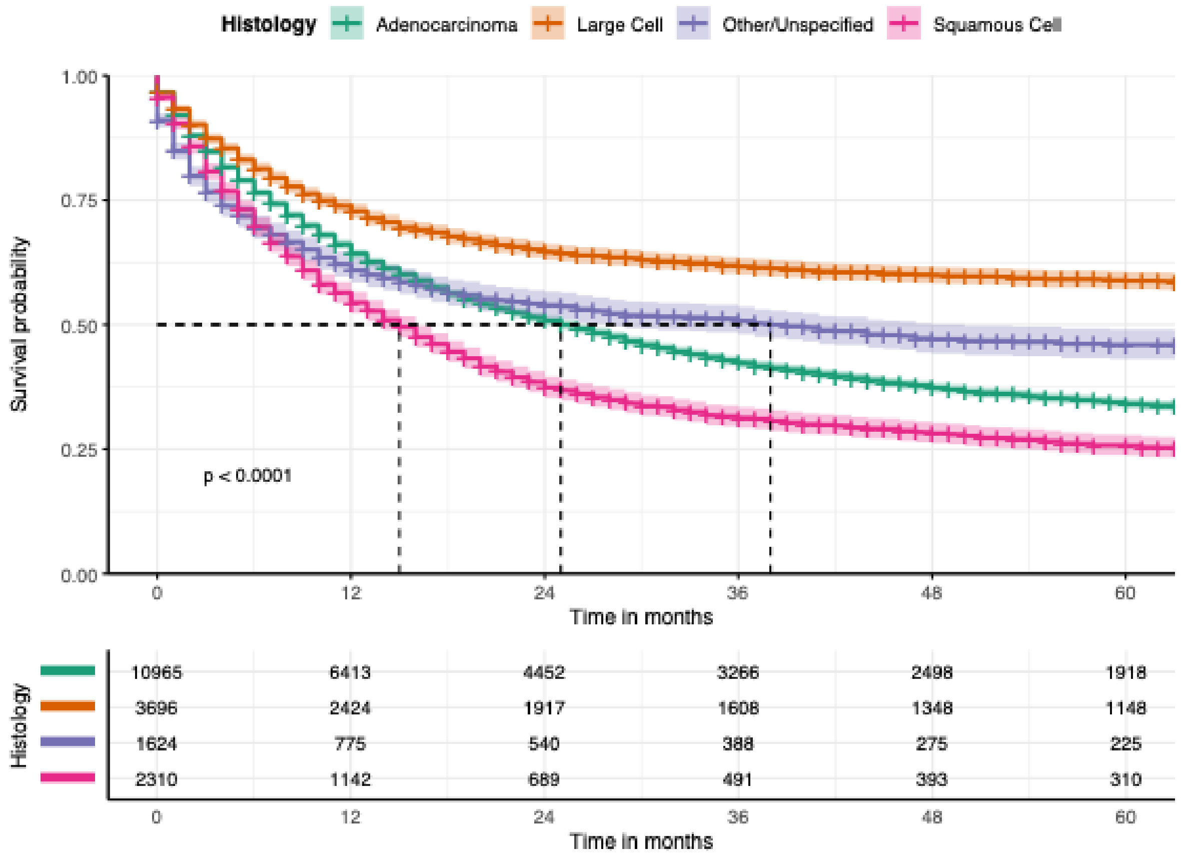

© 2025 Kar i et al. *JAMA Network Open*.

eFigure 6. Global Feature Importance Ranked by Mean Absolute SHAP Values From the Random Survival Forest Model

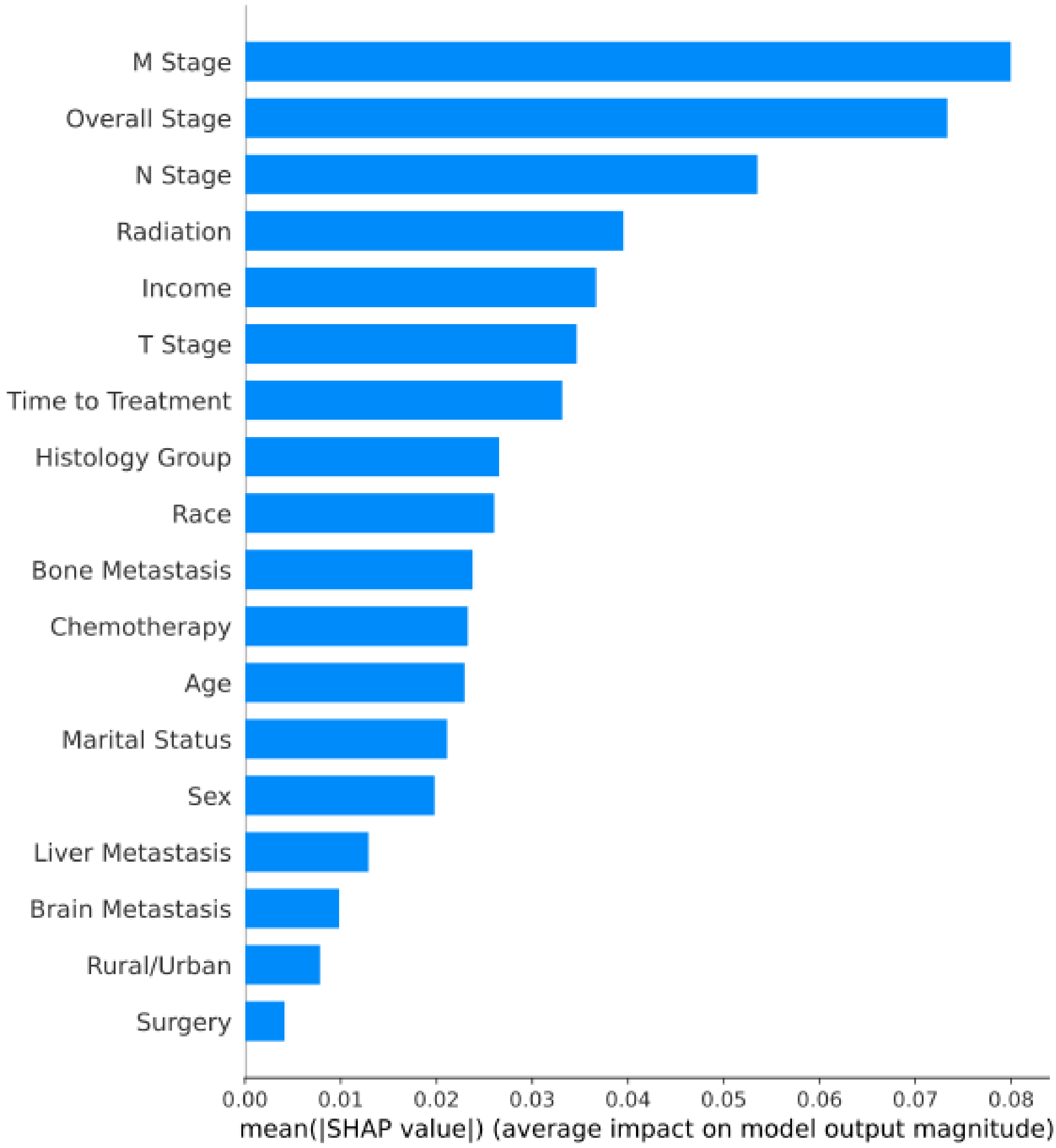

© 2025 Kar i et al. *JAMA Network Open*.

eTable 2. Number of Patients and Events by Treatment Group and Overall Stage

	Time to Dx to Tx	Number of Patients	Event, No. (%)
**Stage 1**	**≤2w**	1,189	100 (8.4)
	**>2w-≤4w**	488	43 (8.8)
	**>4w-≤6w**	448	55 (12.3)
	**>6w-≤8w**	319	30 (9.4)
	**>8w**	618	58 (9.4)
**Stage 2**	**≤2w**	321	78 (24.3)
	**>2w-≤4w**	203	60 (29.6)
	**>4w-≤6w**	181	48 (26.5)
	**>6w-≤8w**	143	48 (33.6)
	**>8w**	246	52 (21.1)
**Stage 3**	**≤2w**	963	525 (54.5)
	**>2w-≤4w**	520	283 (54.4)
	**>4w-≤6w**	489	244 (49.9)
	**>6w-≤8w**	278	143 (51.4)
	**>8w**	432	222 (51.4)
**Stage 4**	**≤2w**	3,726	2,875 (77.2)
	**>2w-≤4w**	1,954	1,406 (72.0)
	**>4w-≤6w**	1,245	871 (70.0)
	**>6w-≤8w**	568	393 (69.2)
	**>8w**	711	481 (67.7)

Event denotes death attributable to NSCLC. Values are shown as number (%) within each subgroup.

© 2025 Kar i et al. *JAMA Network Open*.

eFigure 7. Kaplan-Meier Survival by Rural vs Urban Residence Across Stages I-IV

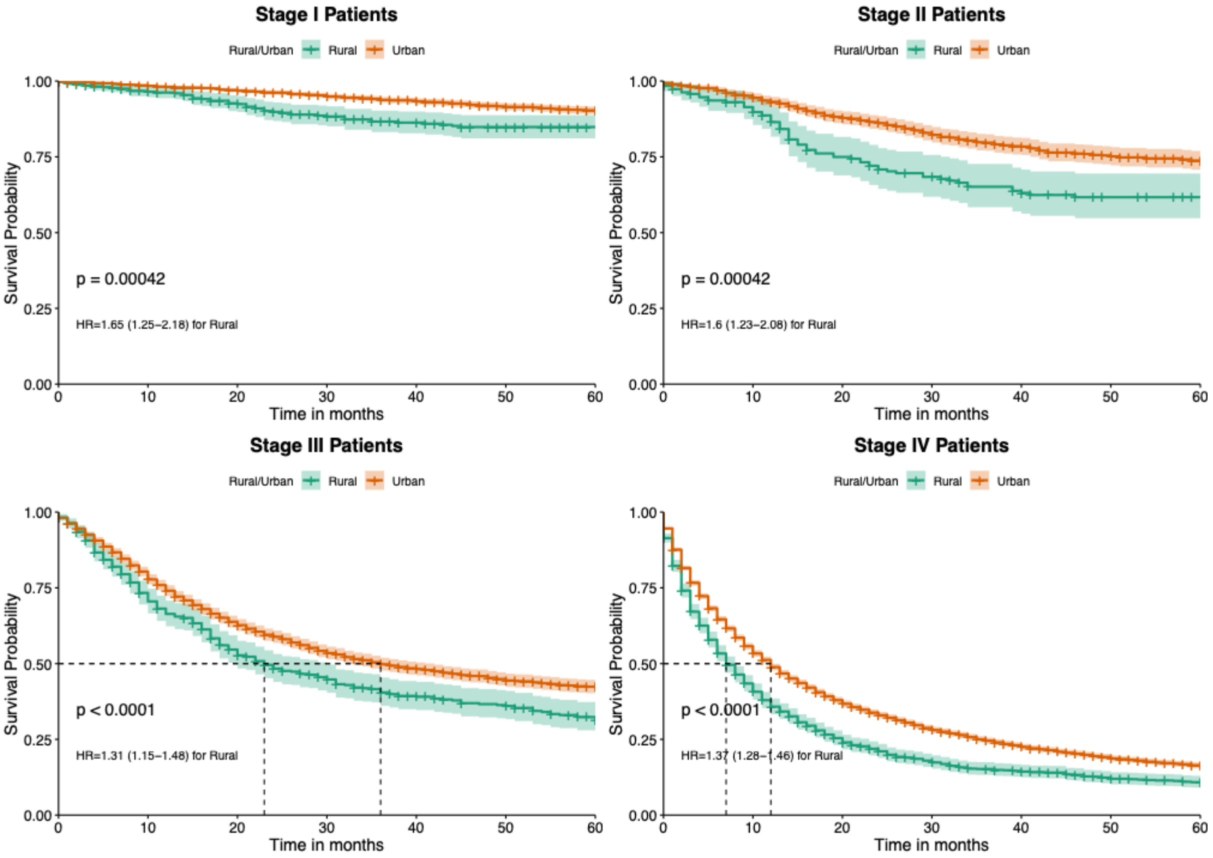

© 2025 Kar İ et al. *JAMA Network Open*.

eFigure 8. Kaplan-Meier Survival by Income Group Across Stages I-IV

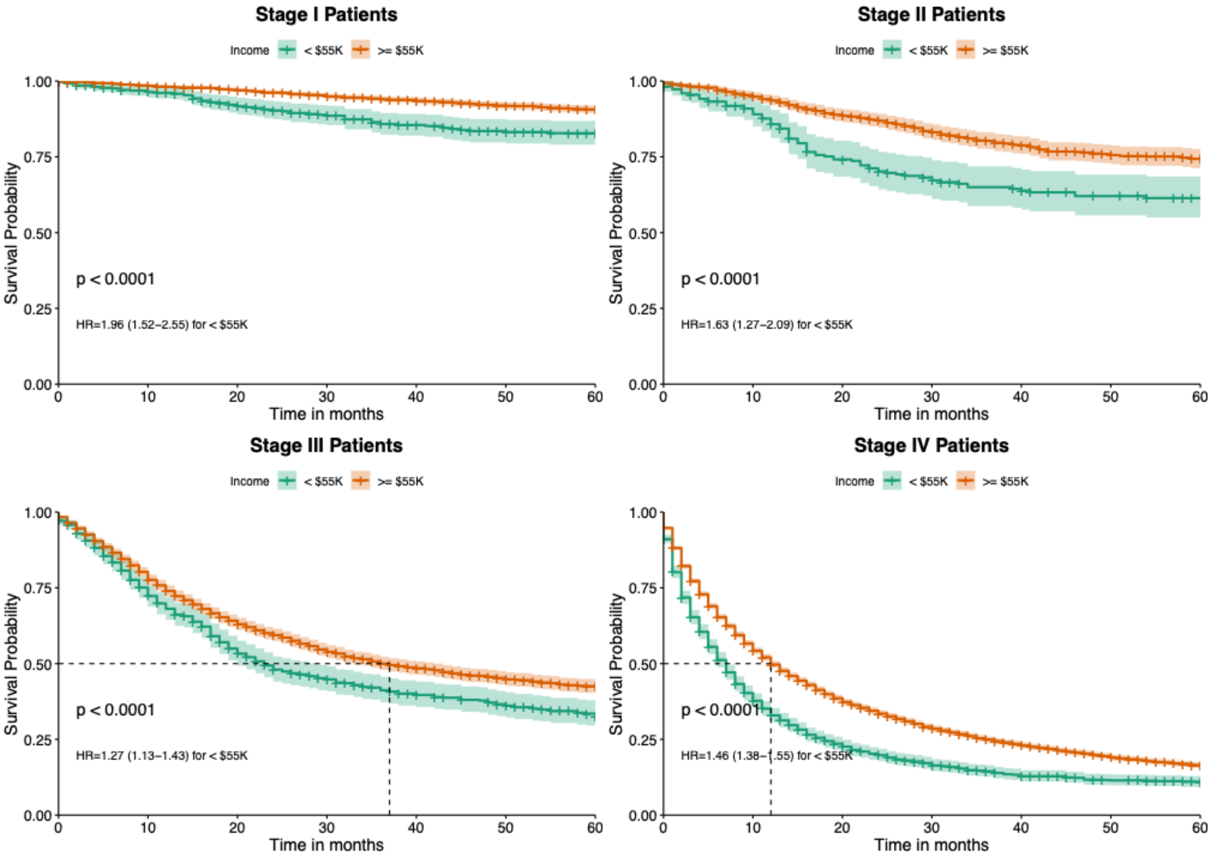

© 2025 Kar i et al. *JAMA Network Open*.
