# Supplementary material for: Survival Determinants and Sociodemographic Disparities in Early-Onset Non–Small Cell Lung Cancer
Source: JAMA Netw Open. 2025 Oct 13;8(10):e2537307. doi: 10.1001/jamanetworkopen.2025.37307 (PMC12519311; doi:10.1001/jamanetworkopen.2025.37307)
Supplement: Supplement 2. — Data Sharing Statement [file jamanetwopen-e2537307-s002.pdf]

Data Sharing Statement

Kar. Survival Determinants and Sociodemographic Disparities in Early-Onset Non–Small Cell Lung Cancer. *JAMA Netw Open*. Published October 13, 2025.
doi:10.1001/jamanetworkopen.2025.37307

Data
Data available: No
